# Perceptions of Resident Autonomy in Internal Medicine, Pediatrics, and Internal Medicine-Pediatrics

**DOI:** 10.7759/cureus.13805

**Published:** 2021-03-10

**Authors:** Alexandra E Mieczkowski, Alda Maria R Gonzaga, Kevin Kraemer, Robert Habicht, Allen R Friedland, Doris Rubio, Reed Van Deusen

**Affiliations:** 1 Division of General Internal Medicine, University of Pittsburgh Medical Center, Pittsburgh, USA; 2 Division of General Academic Pediatrics, University of Pittsburgh Medical Center, Pittsburgh, USA; 3 Department of Medicine, University of Maryland Medical Center, Baltimore, USA; 4 Department of Pediatrics, University of Maryland Medical Center, Baltimore, USA; 5 Department of Medicine-Pediatrics, ChristianaCare, Newark, USA; 6 Department of Medicine, University of Pittsburgh Medical Center, Pittsburgh, USA

**Keywords:** resident, trainee, autonomy, internal medicine, pediatrics, med-peds, im-peds, inpatient

## Abstract

Background: Although graduated autonomy is an essential component of residency training, we have an incomplete understanding of resident and attending faculty perceptions of autonomy.

Objective: In this study, we assessed differences in perceived autonomy among residents and faculty in pediatrics, internal medicine, and combined internal medicine-pediatrics. Methods: We surveyed senior-level (PGY-2-5) residents and faculty in pediatrics, internal medicine, and combined internal medicine-pediatrics in three large, urban training centers in November 2014. The survey included domain items such as general perceptions of autonomy, case management, rounding structure, and individual resident and faculty factors that may interplay with the granting or receiving of autonomy. Results: Of 489 eligible respondents, 215 (44%) responded. Internal medicine-pediatrics residents were more likely than categorical pediatrics residents and pediatrics faculty to disagree that they received an appropriate level of autonomy while on inpatient pediatrics general wards (mean = 2.7 relative to 4.0 and 4.3, categorical residents and faculty; 5-point Likert scale; P < .001). On a 5-point Likert scale, the internal medicine-pediatrics residents were more likely to agree that they received too much oversight on pediatrics general ward rotations (mean, 3.9) compared to internal medicine general ward rotations (mean, 1.9) with a *P-*value between rotations of <.001. Combined internal medicine-pediatrics perceptions of too much oversight while on pediatric general ward rotations were significantly different from their categorical pediatrics peers (pediatrics mean 2.0, P* *< .001). Conclusions: Internal medicine-pediatrics residents have differing perceptions of autonomy from their categorical peers as well as categorical supervising faculty. Combined Internal medicine-pediatrics residents' perceived oversight on pediatrics rotations differently from their categorical pediatrics peers and also differently from their experiences on internal medicine wards. A better understanding of combined internal medicine-pediatrics residents' perceptions of autonomy and supervision can help inform future work regarding autonomy-supportive strategies to optimize learning.

## Introduction

Graduated autonomy is an essential component of residency training across medical specialties [1]. Clinicians and educators are tasked with providing an environment in which patients receive safe, effective, high-quality care, but which also fosters the development and independence of the next generation of physicians.

Graded autonomy interplays in the provision of care and teaching of trainees, as well as educational milestones within the core competencies, construct for both pediatrics and internal medicine (IM) [2,3]. Previous research suggests that a perceived optimal level of autonomy may result in fewer medical errors as well as higher support for patient autonomy [4-6]. In addition, an autonomy-supportive learning environment is more likely to promote self-directed learning and lead to enhanced medical knowledge and skills [7,8]. Mismatches in autonomy perception in the resident-attending dyad may result in dissatisfaction with the learning environment, decreased learning, and increased trainee stress in the clinical environment [4].

Resident and faculty perceptions of autonomy and supervision have been assessed within various individual specialties [9,10]. We previously assessed combined internal medicine-pediatrics (MP) residents’ perceptions of autonomy on each specialty, which revealed perceptions of less autonomy while on pediatrics general ward rotations [11]. The discrepancy between residents’ perceptions of generalist ward months on the two specialties was concerning; however, could not be examined within the broader context of both IM and pediatrics physician perspectives. To our knowledge, no study has assessed MP residents' perceptions of autonomy on inpatient rotations relative to categorical IM and categorical pediatrics peers and faculty. Therefore, we chose to survey residents and faculty to assess perceptions of resident autonomy in these groups. We hypothesized that residents (both pediatrics and MP) on pediatrics rotations would perceive less autonomy relative to residents (both IM and MP) on IM rotations. In addition, we hypothesized that faculty perceptions of autonomy would not differ across specialties.

## Materials and methods

We approached institutions with residency training programs in our areas of interest-categorical IM, categorical pediatrics, and combined MP. Program directors for each of the three training programs at University of Pittsburgh Medical Center (UPMC), University of Maryland Medical Center, and ChristianaCare Health System/ Nemours Alfred I. duPont Hospital for Children agreed to participate and to assist with site coordination.

Survey instrument

We conducted an online survey modeled after a 2014 study examining residents’ perceptions of autonomy, adapted for use with combined and categorical trainees as well as faculty (Appendix) [11]. Several questions related to residents’ and faculty members’ general perceptions of autonomy on the general inpatient wards. It is possible that faculty afford autonomy with respect to which type of resident they were training (Categorical vs MP). For example, an MP faculty member might have a positive bias towards MP residents and thereby afford them more autonomy than a categorical resident. To control for this, we asked faculty to compare the levels of autonomy they gave to categorical vs. Med-Peds residents. Respondents were asked to rate their perceptions of excessive or inadequate attending oversight. We also asked residents to rate their discomfort with making a decision on their own and faculty to rate their discomfort with a resident making a decision on their own. The survey was pilot tested by faculty and chief residents.

Survey administration and participants

The survey was administered via the online survey system Qualtrics (Provo, UT), and participants received surveys tailored for their specific specialty. Categorical IM and pediatrics residents completed a survey with questions regarding either perception of the inpatient IM or pediatrics ward experiences, respectively. MP residents completed a survey containing identical question blocks for both specialties. The block that a combined-specialty trainee received first was randomized in an attempt to mitigate direct comparisons between the specialties. 

All faculty respondents were asked to provide information about the specialty or specialties in which they trained and were practicing. Faculty members then provided a survey appropriate to both their training and current practices. As with the resident survey, if a combined faculty member attended on both specialties, the IM and pediatrics blocks were randomized for which block a respondent received first. Participating programs provided potential respondent e-mail addresses. Individual secure links were created so respondents could only answer the survey one time from that specific link. Residents were deemed eligible to participate if they were postgraduate year 2 (PGY-2) or above in IM, pediatrics, MP, or other combined programs. Faculty was eligible to participate if they attended at least four weeks per year on the generalist inpatient service. One participating site had a policy of not providing e-mail addresses for surveys. For this program, individual use passcodes for eligible respondents were created so respondents could log into the survey system from a forwarded link. The e-mailed links were sent three times at one-week intervals in November 2014. Responses were collected anonymously. No site-specific information was collected. Respondents who completed the survey were offered entry into a raffle for one of several $20 gift cards.

Analysis

Data were analyzed using Stata version 13.1 (College Station, TX). Comparisons between groups were assessed using Wilcoxon rank-sum and chi-square tests, as appropriate. Medians, means, and interquartile ranges are provided.

The study was deemed exempt by the University of Pittsburgh and University of Maryland institutional review boards. The programs within ChristianaCare Health System and Nemours Alfred I. duPont Hospital for Children did not require additional approval from their institutional review boards.

## Results

Response rates

Survey invitations were sent to a total of 489 potential respondents, including 351 residents and 138 faculty members. Of 489, 215 (44%) responded. The resident response rate was 40% and the faculty response rate was 52%. MP residents and faculty had the highest response rates at 89% (29/35) and 75% (8/12), respectively (Table 1). Of resident respondents, 41% (55/133) were PGY-2, 50%(66/133) were PGY-3, and 9% (12/133) were PGY-4 and above, which was similar to the PGY levels for eligible respondents. Total demographic numbers are lower than total resident respondent numbers because respondents were not required to answer all demographics questions. Pediatrics residents were more likely to identify as female than MP or IM residents (P < .001); however, respondents’ gender was similar to those of potential respondents.

**Table 1:** Survey Response Rates by Specialty and Type Response rates are the total respondent in the category by an overall eligible number of respondents in the category

	Internal Medicine	Pediatrics	Combined MP	Total
	n/N (%)			
Residents	63/171 (37)	50/145 (34)	29/35 (89)	142/351 (40)
Faculty	43/83 (52)	21/43 (49)	8/12 (75)	72/138 (52)
Total	106/254 (42)	71/188 (38)	37/47 (79)	214/489 (44)

General perceptions of autonomy

**Figure 1:**
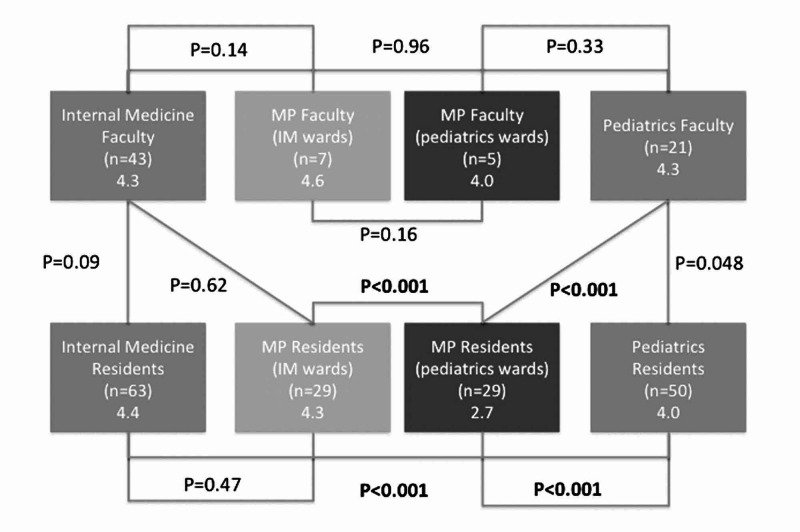
Overall Perceptions of Autonomy in Inpatient Internal Medicine and Pediatrics General Wards “Overall during the course of the past year, my attendings gave me an appropriate level of autonomy”. “Overall during the course of the past year, I gave my residents an appropriate level of autonomy". Responses represent resident and faculty respondents asked generally about autonomy in the inpatient ward setting. Lines with P-values denote comparisons between groups surveyed. IM groups are to the left of the figure. Pediatrics groups are to the right of the figure. 1 = Strongly Disagree, 2 = Disagree, 3 = Neutral, 4 = Agree, 5 = Strongly Agree. Box (specialty, respondents, mean). P-values, Wilcoxon rank-sum testing

IM, pediatrics, and MP faculty attending on both specialties either strongly agreed or agreed that residents receive an appropriate level of autonomy (Figure 1). Categorical IM residents and pediatrics residents either strongly agreed or agreed that they received an appropriate level of autonomy (median = 5 and 4, interquartile range [IQR] 4-5 and 4-4, respectively). MP residents strongly agreed that they received an appropriate level of autonomy while on inpatient IM general wards (median = 5, IQR 4-5), and disagreed that they received an appropriate level of autonomy while on inpatient pediatrics wards (median = 2, IQR 2-3). For pediatrics general ward rotations, MP residents were significantly more likely to disagree that they received an appropriate level of autonomy compared to their own responses for IM wards (P < .001), and compared to pediatrics residents’ responses (P < .001) and pediatrics attendings’ responses (P < .001). While pediatrics residents generally agreed that they received an appropriate level of autonomy, they differed significantly from their attending physicians, who agreed more strongly that the level of autonomy was appropriate (P = .048). There was no significant difference among IM residents, MP residents on IM rotations, and IM faculty responses. Resident responses were similar by PGY level with no statistically significant differences.

Categorical pediatrics and IM faculty both responded that categorical and MP residents would be given the same level of autonomy. There were few MP faculty attending pediatrics (n = 5), but they were significantly more likely to give MP residents more autonomy (P = .002) than categorical residents.

MP residents on IM wards, and both IM and pediatrics categorical residents, disagreed that there was too much oversight on inpatient wards (Table 2). MP residents, however, agreed that there was too much oversight on general pediatrics wards, and these responses were significantly different compared to their responses for IM wards and compared to their categorical pediatrics peers (P < .001 for both). There were no significant differences between MP respondents on IM wards and their IM peers. Though overall pediatrics residents disagreed that there was too much oversight, their responses were significantly different from the IM resident responses (IM mean = 2.19; pediatrics mean = 2.68; P = .003).

**Table 2:** Resident Perceptions of Oversight From Attending Faculty Response categories: 1 = Strongly Disagree, 2 = Disagree, 3 = Neutral, 4 = Agree, 5 = Strongly Agree. Median responses presented. Wilcoxon rank-sum tests used to compare between groups. Non-significant P-values not shown. ^a^IQR = interquartile range

Survey Item	Internal Medicine (IM) Residents (n = 63)	Medicine-Pediatrics (MP) Residents (n = 29)		Pediatric Residents (n = 50)
		IM Rotation	Pediatrics Rotation	
During the course of the past year, I believe that there was TOO MUCH oversight by attendings	2.2 (IQR 2-2)^a^ P = .08 vs MP (IM); P = .003 vs pediatrics	1.9 (IQR 2-2) P < .001 vs MP pediatrics rotation	3.9 (IQR 4-4) P < .001 vs pediatrics	2.7 (IQR 2-3)
During the course of the past year, I believe that there was TOO LITTLE oversight by attendings	2.1 (IQR 2-2) P = .003 vs MP (IM)	2.5 (IQR 2-3) P < .001 vs MP pediatrics rotation	1.6 (IQR 1-2) P = .002 vs pediatrics	2.0 (IQR 2-2)

Faculty in all specialties disagreed that residents expected too little oversight (median = 2) (Table 3). Most faculty members also disagreed that residents expected too much oversight. Pediatrics faculty members were overall neutral (mean = 3.0); however, they were significantly more likely than IM faculty (mean = 2.4) to agree residents expected too much oversight (P = .03).

**Table 3:** Faculty Perceptions of Resident Expectations of Oversight Response categories: 1 = Strongly Disagree, 2 = Disagree, 3 = Neutral, 4 = Agree, 5 = Strongly Agree. Median responses presented. Wilcoxon rank-sum tests used to compare between groups. Non-significant P-values not shown. ^a^IQR = interquartile range

Survey Item	Internal Medicine Faculty (n = 43)	Medicine-Pediatrics (MP) Faculty (IM) (n = 7)	MP Faculty (Pediatrics) (n = 5)	Pediatric Faculty (n = 21)
During the course of the past year, I believe my residents expected TOO MUCH oversight	2.4 (IQR 2-3)^a^ P = .03 vs pediatrics	2.6 (IQR 2-4)	3.4 (IQR 2-4)	3.0 (IQR 2-4)
During the course of the past year, I believe my residents expected TOO LITTLE oversight	2.2 (IQR 2-2)	2.4 (IQR 2-3)	2.6 (IQR 2-2)	2.3 (IQR 2-3)

Discomfort with decision-making

In order to assess respondents' sense of autonomy on clinical decision-making, we asked residents to report discomfort with making decisions without attending input. On a 6-point Likert scale (1 = Never to 6 = Always), MP residents reported discomfort making decisions without attending input more often while on pediatrics rotations (mean = 3.3) compared to IM (mean = 2.8, P = .01), but they were comparable to both their pediatrics (mean = 3.1) and IM peers (mean = 2.7). MP residents (mean = 3.3) reported more discomfort with making a decision than pediatrics faculty (mean = 2.6) felt in the resident making that decision (P = .006), and a similar relationship was seen between categorical pediatric residents and faculty (P = .006). Categorical residents, faculty, and MP residents on IM rotations had similar comfort levels with a resident making decisions without attending input.

## Discussion

Our results reveal significant differences between MP residents’ perceptions of autonomy and those of their categorical IM and pediatrics peers. In line with our initial hypothesis, MP residents perceived less autonomy and too much oversight while on pediatrics general inpatient ward rotations. Additionally, MP residents’ general perceptions of autonomy often differ significantly from the pediatrics attendings’ (but not IM attendings’) perceptions. Both MP and pediatrics residents were more uncomfortable with making autonomous decisions, despite pediatrics attendings' higher level of comfort with them doing so, revealing a potential mismatch in expectations about autonomy. We hypothesized that faculty perceptions would be similar across specialties; however, some differences were noted. While we previously understood that MP residents perceived less autonomy on pediatrics wards relative to IM wards rotations, we can now place MP residents’ perceptions within the context of the perceptions of their categorical peers and faculty.

There may be several reasons for the observed differences. First and foremost, MP residents are in the unique situation of being able to compare experiences on two distinct specialties. Literature suggests that there has been an “erosion” of autonomy within the specialty of pediatrics in at least one training program, and, with comparative experience in two specialties, our MP residents’ responses may serve as a bellwether that autonomy may be a concern in other programs as well [12,13]. The differences in perception may stem from pediatrics faculty having less experience with MP residents who only spend half their time with each specialty; however, MP residents may also be less known to IM faculty, and the same differences in perception were not seen.

Despite both being primary care specialties, the culture of care in dealing with disparate patient population ages may vary from pediatrics to IM, in which the expectations for faculty presence and supervision may therefore appropriately differ. However, supervision and autonomy may coexist and have a positive impact on each other since perceptions of increased supervision may actually improve resident reported autonomy [14].

Our study has several strengths. We surveyed MP residents in addition to their categorical counterparts, offering a unique perspective of autonomy and supervision. Given the need to assess more than residents’ perspectives, we also surveyed faculty members who are often the other key player in the autonomy-supervision dyad. Finally, we used a multi-site approach, which broadened the generalizability of our results.

However, there are also limitations. Despite a robust response rate from MP respondents generally, our overall response rate for categorical respondents was relatively low. We attempted to address generalizability by utilizing three separate sites; however, our sites were clustered in the mid-Atlantic region. Finally, our study assessed resident and faculty perceptions of autonomy. Respondents’ perceptions may differ from the supervision relationships viewed through the lens of an impartial observer.

## Conclusions

There are significant differences in the perception of autonomy in the two primary care specialties of pediatrics and IM. These seem to be noted most by MP residents who experience the general inpatient wards of both specialties, but also noted in some respects by categorical pediatrics residents. Faculty development programs aimed at teaching and enhancing skill sets specific to autonomy-supportive supervision may be an initial starting point toward an optimal experience for both residents and faculty.

## Appendices

**Table 4:** Survey Instrument Items PGY - postgraduate year

Resident and Faculty Items	Resident Only Items	Faculty Only Items	Combined Resident/Faculty Only Items
Overall Autonomy	PGY Level	Subspecialty Training	Current specialty (Resident only)
Too Little Oversight	Prepared for Practice (Senior resident only)	Years in Practice	Specialties attending (Faculty only)
Too Much Oversight	Age	Med-Peds residents (compare autonomy to categorical residents)	
Discomfort with decision-making			
Gender			
